# A special case of acute leukemia in childhood

**Published:** 2011-08-25

**Authors:** ID Bleahu, R Vladasel, A Gheorghe

**Affiliations:** * ‘Fundeniߣ Clinical Institute, ‘Carol Davila’ University of Medicine and Pharmacy Pediatrics Department Romania; ** ‘Fundeni’ Clinical Institute, Pediatrics Department Romania; *** ‘Fundeni’ Clinical Institute, Hematology DepartmentRomania

**Keywords:** bilineage leukemia, biphenotypic leukemia, children

## Abstract

Hybrid leukemia is a clinical entity that includes: biphenotypic leukemia, characterized by the presence of markers of more than two lineages of a single tumor cell, bilineage leukemia, a combination of more than two lineage markers on two distinct blast cells, and biclonal leukemia, the concomitancy of more than two types of leukemic cells, derived from different clonal expansions. We present a case of a 7–year–old female diagnosed with bilineage leukemia. We propose a treatment for biphenotypic/bilineage leukemia in the cases with good prognostic factors. We suggest that hematopoietic stem cell transplantation is often not required for cure of these patients.

## Introduction

The diagnostic criteria for the classification of acute leukemia include: morphological appearance, immunophenotype blasts, cytogenetic and molecular analyses. The examination of the bone marrow aspirate will allow, in most of the cases, to distinguish the lymphoblasts from the myeloblasts. Various cytochemical stains can distinguish lymphoblasts from myeloblasts: the lymphoblasts react positively with periodic acid–Schiff (PAS), with stains cytoplasmic glycogen, and the myeloblasts are myeloperoxidase positive in 75% of the patients.

Fab ( French–American–British) morphological classification divides acute lymphoblastic leukemia (ALL) in three types (L1 , L2, L3) and nonlymphoblastic acute leukemia in eight types (M0 to M7). For acute lymphoblastic leukemia, this classification, except for the L3 type (Burkitt) identification, has lost its importance.

The immunophenotyping of the leukemic blasts is essential to distinguish between B and T lymphoblastic line (Table 1). The immunological markers CD19, CD20, CD22, CD79a belong to line B, while CD2, CD3, CD5, CD7 are found on the surface of the T cells.

Except for M0 and M7 (CD41, CD61), the myeloid line markers are: CD13, CD33, CD15, CD117 and also the myeloperoxidase (MP0). According to the classic definition, acute leukemia is considered a malignant clonally proliferation of the hematopoietic precursors, on one line and so the blast cells frequently express markers of a single cell line (lineage fidelity). In a variable number of cases, an aberrant antigen expression can be found (lineage infidelity).

**Table 1:** Immunological classification of acute lymphoblastic leukemia; cIg= intracytoplasmic immunoglobulin; cyt CD22= cytoplasmic CD22; sIg= surface immunoglobulin

B line	
Pro–B	CD19+ and/or CD79a+ and/or CD22 cytoplasmic +, CD10–, cIg–
Pre B	cIg +, CD19+, CD20+, CD24+, CD22+CD10+ HLADR+
B mature	CsIg+ cIg–, Express other B–cell antigens including CD19, CD20, CD24 HLADR
T line	
Pre T	CD7+( pro–T) , Cd2+,CD5+,CD8+(pre–T)
T mature	CD1a + ( T cortical ), CD35+,CD1a– ( Tmature)

This appears as the expression of one or more markers that belong to different cell lines. These cases were described as ALL expressing myeloid antigens (ALL My+) or acute myeloid leukemia (AML) with lymphoid markers (AML Ly+). More studies on these cases have shown that the lineage infidelity has no prognostic significance [1].

### Mixed acute leukemia (ambiguous lineage leukemia)

In contrast to acute leukemia with lineage infidelity, the ambiguous lineage leukemia (hybrid leukemia) is a rare and heterogeneous group of acute leukemia, which presents the characteristics of both myeloid and lymphoid precursors [2]. Divergent morphological and immunophenotype features can be presented in a single blast population (biphenotypic leukemia) or in two morphologically distinct populations (bilineage leukemia). Cases of acute leukemia with lineage switch at relapse (from ALL to ALM or vice versa) have been reported as well. These forms can be considered as particular cases of acute bilineage leukemia [3,4]. The leukemic transformations occur in both cell lines, however, there is only one line expressed at diagnosis. In the case reported by Imatuki and col, the bone marrow went into remission after induction chemotherapy but the residual disease in the patient's liver caused another phenotypic leukemia to arise [3]. The leukemogenic additional event would be the chemotherapy treatment itself. It has been postulated that a multipotent stem cell is capable of differentiating between more than one lineage. This feature of leukemic cells is described by the term ‘cancer stem cell’. The classification of ambiguous lineage leukemia is presented in (Table 2) (according to the World Health Organization). The difference between acute biphenotypic leukemia and an ALL / AML with lineage infidelity is made through EGIL score (European Group for the Immunological Characterization of Leukemia), presented in (Table 3) [5]. To define a veritable biphenotypic acute leukemia the markers' score on a least two lines must be greater than 2.

**Table 2:** Classification of ambiguous lineage leukemia (according to World Health Organization)

undifferentiated acute leukemia	Absence of specific markers of myeloid or lymphoid line
Mixed acute leukemia with t (9;22) / bcr–abl	Mixed phenotype with translocation (9;.22) or bcr–abl fusion
Mixed acute leukemia with 11q23 rearrangements	Mixed phenotype with MLL gene rearrangements
Mixed acute leukemia B / myeloid	Mixed phenotype with B line and myeloid markers without bcr–abl fusion or MLL gene rearrangements
Mixed acute leukemia T / myeloid	Mixed phenotype mixed with T and myeloid markers without bcr–abl fusion or MLL gene rearrangements
Mixed acute leukemia T/B/ myeloid	Mixed phenotype with T, B and myeloid markers
Mixed acute lymphoblastic leukemia T / B	Mixed phenotype with B and T markers
Other	natural killer cell lymphoma/leukemia

**Table 3:** Immunological score (European Group for the Immunological Characterization of Leukemia)

Score	B line	T line	Myeloid line
2 points	CD79a CD22 IgM cyt	CD3 Anti TCRαβ Anti TCRγδ	Anti–MPO
1 point	CD19 CD10 CD20	CD2 CD5 CD8 CD10	CD13 CD33 CD117 CDw65
0,5 points	TdT CD24	TdT	CD14 CD15 CD64

Biphenotypic acute leukemia (BAL) occurs both in children and adults, but epidemiological data are rare. In a retrospective epidemiological study conducted on 693 adults and children cases with acute leukemia [6] BAL were detected in only 25 cases (3,6%). Amal and al reported a 4,3% frequency of  biphenotypic leukemia in a study in children with acute leukemia [1]. The frequency in children seems to be less than in adult.

Similarly to AML cases, acute leukemia with ambiguous lineage may occur de novo or may be secondary to chemotherapy and/or radiotherapy. The morphological appearance of blasts is heterogeneous (in one third of the cases there are lymphoblasts, and almost two thirds of cases are myeloblasts), very rarely there are two distinct populations. In cases with AML appearance, the M3, M6 and M7 types are exceptional, if not nonexistent [8]. According to the EGIL immunological score, several subgroups of BAL have been identified, namely: BAL with B lymphoid and myeloid markers coexpression (B/M), BAL with lymphoid markers T and B (T/B) and BAL with the coexpression of T/B/ myeloid [7]. The most frequent myeloid markers are CD33, CD13, CD11b. The most common forms are the BAL B/M, followed by BAL T/M.  BAL T/B is rare and BAL T/B/M is exceptional [8]. The cytogenetic and molecular analysis is essential for the prognosis and for the therapeutic attitude in all leukemia cases. The most common cytogenetic anomalies in BAL are t (9,22)(q34;q11), called the Philadelphia chromosome (Ph1) or bcr/abl rearrangements (type p190) and the rearrangements of 11q23 band (frequently observed in infants with ALL). Translocation (9,22) leads to the loss of regulatory domain of ABL tyrosine kinase which makes changes in the activity of multiple pathways involved in cellular differentiation [9] . The MLL gene (11q23) encodes a large complex oncoprotein that regulates transcription. MLL (mixed lineage leukemia) maintains HOX gene expression during hematopoiesis.

MLL translocations disrupt a breakpoint cluster region and fuse the 5' portion of MLL with one of more than 50 different partner genes generating diverse leukemogenic fusion oncoproteins.

Philadelphia chromosome is more common in adult cases, versus pediatric age (5%), but the frequency of t (9;22) in pediatric BAL is not documented. In a study of 35 children with BAL, Rubnitz and al reports the presence of an abnormal cariotype in only 29 of the 33 studied cases. MLL gene rearrangements are observed in 4 cases and were not otherwise identified if not for recurrent abnormalities, thus t (9;22)(10).

In contrast to t (9,22), rearrangements of  the TEL–AML1 or t (12,21) are common in children and associate a favorable outcome. TEL–AML1+ patients have an increased expression of CD13 and CD33 compared with those who are TEL–AML1 negative. Over 60% of the children BAL have a TEL–AML1 rearrangement compared to only 25% of pediatric ALL.

### Prognosis

Older studies reported a poor prognosis and a long–term survival rate of only 8% [2]. This would require a more intensive treatment with high doses and allografts. On the other hand, these studies mostly include adults with a high percentage of Ph1 cases. The situation is different in pediatric groups. Killick and al report a 2 year survival rate of 39% for all cases (adults and children), but six of the eight children with BAL were in remission at 2 years after treatment. The authors have suggested that the prognosis in children with BAL does not differ from other forms of acute leukemia.

The bilineage acute leukemia has a much lower frequency than biphenotypic acute leukemia, that is 1% of all cases (adults and children) and also a long–term remission was achieved in two of the 16 studied cases. In a study from St.Jude Children's Hospital, over a period of 20 years, we only identified 35 cases of BAL in children (2%), and, out of these, 49% are long term survivors. The analysis of cases demonstrates the difficulty of prediction in patients who respond better to the chemotherapy protocol for ALL versus AML. Rubnitz and al suggest that the stem cell transplantation should only be applied in cases with a percentage of marrow blasts over 1% after induction. For cases with a rate of blasts below 1%, the choice of treatment is more difficult [10].

## Case report

A 7–year–old female child was admitted to our unit on October 12, 2005 for the insidious onset of pallor and fatigue. On admission, the clinical picture showed pallor and moderated hepatosplenomegaly, without hemorrhagic manifestation. Blood cell count showed normochromic normocytic anemia (hemoglobin 7,4g/dl), moderate leukocytosis (16.000/μl) and moderate thrombocytopenia (82.000/ μl). Peripheral blood smear revealed 30% blast cells with morphologic appearance of L2 lymphoblasts. The hepatorenal, inflammatory and haemostatic balance was within normal limits. The cerebrospinal fluid examination was normal. The microscopic examination of the bone marrow aspirate showed a high cellular marrow with three distinct blast populations: myeloblasts (50%), promyeloblasts (10%) and L2 type lymphoblasts (20%). Flow cytometry revealed the positivity of the following immune markers: CD22+, CD19+, CD10+, CD13+, CD33+, HLA–DR+ and the cytochemical reaction was positive for myeloperoxidase. Chromosome analysis for the blast cells was normal. According to EGIL score, 4 points on each line of B lymphoid and myeloid line were obtained. From the immunological point of view, this case was an acute biphenotypic leukemia B/M and from the morphological point of view, this case was acute bilineage leukemia. In this case, the treatment was a combination of AML and ALL protocol, but with a different maintenance treatment. The treatment doses were calculated per m^2^, and body surface area was of approximately 1 m^2^. The full treatment protocol is shown in (Table 4).

**Table 4:** Treatment protocol in the case report; Doses were calculed for a body surface area of 1 m^2^

Remission induction ( 2 cycles)	Dexametasone: 8mg i.v.; days 1–12(daily) Cytarabine: 100mg days 1–3 and 200mg days 4–9 Vincristine 1,5mg ; day 1 and day 9 Mitoxantrone: 12mg ; days 4– 5 Etoposide : 200 mg ; days 6 –8
Early intensification ( consolidation)	Cytarabine: 75 mg; days 1–4; 8–11; 15–18; 29–32; 36–39; 43–46( 6 cycles) 6–Thioguanine : 60mg(po) ; days 1–46(daily) Vincristine: 1,5mg ; days 1, 15, 29, 43 Mitoxantrone: 12 mg ; days 1, 15, 29, 43
Intensification high–dose cytarabine	Cytarabine: 3g ; days 1, 2, 3 Mitoxantrone :12 mg ; days 3, 4, 5
Reinduction therapy	Dexametasone: 8mg iv ; days 1–33(daily) Vincristine: 1,5mg ; days 8, 15, 22, 29 Daunorubicin : 30mg days 8, 15 ,22, 29 L–Asparaginase : 5000U; days 12, 15, 18,21
Maintenance therapy	6–Thioguanine : 60mg (po); daily for 14 months Cytarabine : 80mg; days1,2,3,4( monthly for 12 months) Cyclophosphamide : 100mg: days 1,2,3,4 (monthly for 12 months)

Remission induction was based on modified French society of oncopediatric protocol (1989). At the end of the first induction, the bone marrow aspirate smears showed a 7% residual blast infiltration. A second induction was started on day 15, using the same treatment, and, at the end of induction, the child was in complete remission (0% marrow blasts). Early intensification (consolidation), immediately after remission induction, belonged to the same protocol with dose adjustment of mitoxantrone (12mg instead of 8mg). The advantage of this treatment was its similarity to the consolidation therapy in childhood ALL treatment (BFM 2002 protocol). Intensification therapy with high–dose cytarabine was consistent with modern protocol for AML. Reinduction therapy belonged to ALL–BFM 2002 protocol. The maintenance cures covered both blast populations. Prophylaxis of the central nervous system (CNS) leukemia was performed during the first year of treatment. Cessation of treatment was made on 01/08/2007. Until now, the infant has remained in complete remission (3 years and 10 months after stopping the treatment). No sequelae of chemotherapy (neuropsychic and somatic development are normal as well as the cardiac and endocrine function) were identified.

## Discussion

Mixed acute leukemia is a rare entity in children. It must be differentiated by acute leukemia with infidelity lineage, as both prognosis and therapeutic approach are different.  Published reports on mixed acute leukemia are limited so it is difficult to estimate the incidence. In the ‘Fundeni’ Clinical Institute, Department of Pediatrics, 219 new cases of acute leukemia were diagnosed between December 2005 and December 2010, of which 6 were BAL cases (2,7%) and  only one of these cases was a bilineage acute leukemia . The pathogeny of hybrid phenotype is complex.  Leukemic transformation occurs at the level of a multipotent progenitor. This progenitor has markers for more cell lines and a genetically diverse mosaic. The appearance of hybrid phenotype can be explained by the fact that the cell preserves some relics of the first attempts at differentiation. The differentiation of a cell line is accompanied by stop signal mechanisms for recombination and antigens expression for the other lines. If the stop signal events do not work, the differentiating process continues to operate simultaneously on different cell lines and thus to a cell clone with the markers that belong to several lines [11]. This lineage ambiguity model does not explain very well the appearance of bilineage leukemia. Is the leukemic transformation in these cases done at the level of a multipotent progenitor or it occurs simultaneously at the two precursor cells?  In other words, BAL is an entity separated from bilineage AL or the two are different expressions of a single process?  In the clinical case, which phenotipically corresponds to BAL B/M , there is a particularity: the presence of the promyelobasts , which in the published data are considered  extremely  rare.  Given the rarity of these cases, unlike the ALL and AML, there are no studies on prognostic factors. In principle, the known prognostic factors (age, hyperleukocitosis, pejorative cytogenetic anomalies, CNS leukemia and the initial response to treatment) can be applied in these cases. Our case is included in the forms with favorable prognosis: age between 1 year and 9 years, absence of pejorative cytogenetic anomalies, hyperleukocitosis and response to treatment. The major problem in these cases is the choice of therapeutic regimes. However, hematopoietic stem cells transplantation should be reserved in cases with t (9,22) or bcr/abl  fusion  and  MLL  rearrangements or in cases with  poor initial  response to treatment. Cases with favorable prognostic factors may only benefit from chemotherapy. The therapeutic line choice is still random, but in the future, a unitary therapeutic protocol might be developed.
